# The molecular basis of hypertrophic scars

**DOI:** 10.1186/s41038-015-0026-4

**Published:** 2016-01-21

**Authors:** Zhensen Zhu, Jie Ding, Edward E. Tredget

**Affiliations:** 1 Wound Healing Research Group, Division of Plastic and Reconstructive Surgery, University of Alberta, Edmonton, Alberta Canada; 2 Division of Plastic Surgery, Department of Surgery, University of Alberta, Edmonton, Alberta Canada; 3 Department of Burn and Reconstructive Surgery, 2nd Affiliated Hospital of Shantou University Medical College, Shantou, Guangdong China

**Keywords:** Hypertrophic scars, Animal model, Cytokines, Growth factors, Macrophages, Stromal cell-derived factor 1/CXCR4 signaling

## Abstract

Hypertrophic scars (HTS) are caused by dermal injuries such as trauma and burns to the deep dermis, which are red, raised, itchy and painful. They can cause cosmetic disfigurement or contractures if craniofacial areas or mobile region of the skin are affected. Abnormal wound healing with more extracellular matrix deposition than degradation will result in HTS formation. This review will introduce the physiology of wound healing, dermal HTS formation, treatment and difference with keloids in the skin, and it also review the current advance of molecular basis of HTS including the involvement of cytokines, growth factors, and macrophages via chemokine pathway, to bring insights for future prevention and treatment of HTS.

## Background

**Fig. 1:**
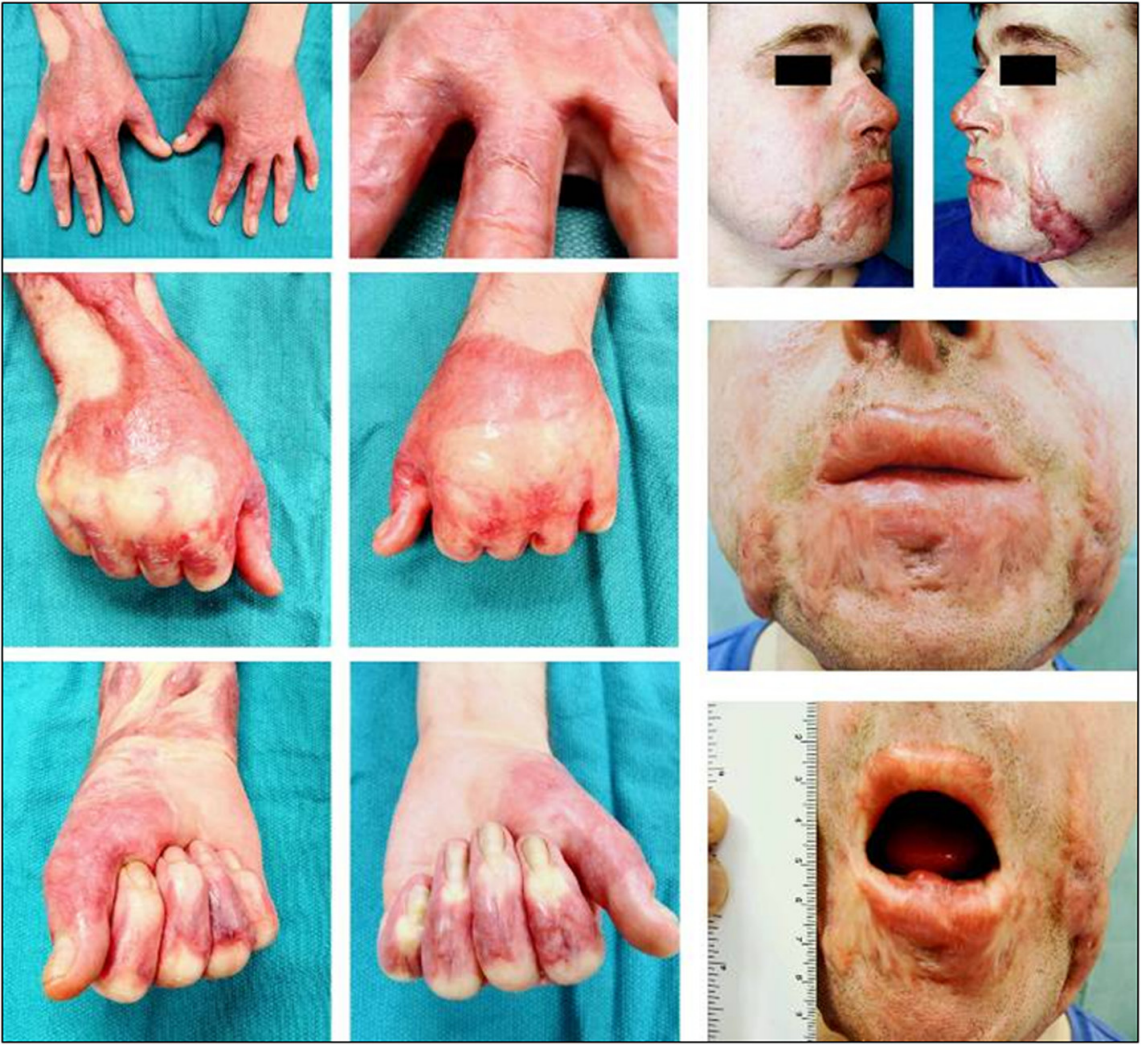
Patients with HTS. A 24 year-old white man, 11 months after a 21 % TBSA burn. This patient developed HTS, resulting in cosmetic and functional problems that included restricted opening of mouth and tight web spaces of fingers that limited range of motion on hands (From Tredget EE, Levi B, Donelan MB. Biology and principles of scar management and burn reconstruction. Surg Clin North Am. 2014 Aug;94(4):793–815. With permission)

Hypertrophic scars (HTS) are considered to be a dermal form of fibroproliferative disorders that are caused by aberrant wound healing due to injuries to the deep dermis, including burn injury, laceration, abrasions, surgery and trauma. HTS are red, raised, rigid and can cause pruritus, pain and joint contracture. HTS formed in the facial area can cause cosmetic disfigurement, which result in psychological and social issues [1, 2] (Fig. 1).

### The physiology of wound healing in the skin

Wound healing can be divided into four stages: hemostasis, inflammation, proliferation and tissue remodeling [3]. In these four stages, there are complicated interactions within a complex network of pro-fibrotic and anti-fibrotic molecules, such as growth factors, proteolytic enzymes and extracellular matrix (ECM) proteins [4, 5].

The first stage is hemostasis, which relates to the clotting cascade and the formation of a provisional wound matrix. These changes occur immediately after injury and are completed within hours [6]. Clotting factors from the injured skin (extrinsic system) and aggregation of thrombocytes or platelets after exposure to collagen fibers (intrinsic system) are activated. The exposed collagen also triggers platelets to begin secreting cytokines and growth factors [7]. The provisional wound matrix serves as a scaffold structure for the migration of leukocytes, keratinocytes, fibroblasts and endothelial cells. Platelets induce the vasoconstriction in order to reduce blood loss followed by secretion of a number of inflammatory factors including serotonin, bradykinin, prostaglandins and most importantly histamine, which activate the inflammatory phase.

In the inflammatory phase, polymorphonuclear neutrophils (PMNs) are the first inflammatory cells that are recruited to the inflamed site and are present there for 2–5 days. Several mediators such as tumor necrosis factor-alpha (TNF-α), interleukin-1 beta (IL-1β) and interleukin-6 (IL-6) are released by the neutrophils in order to amplify the inflammatory response [8]. Monocytes are attracted by the inflammatory mediators and differentiate into macrophages soon after they migrate into the wound site. The main functions of macrophages are phagocytosis of pathogens and cell debris as well as the release of growth factors, chemokines and cytokines which will push the wound healing process into the next stage.

The proliferation stage consists of angiogenesis, re-epithelialization, and granulation tissue formation. The process of angiogenesis is commenced by growth factors such as vascular endothelial growth factor (VEGF) released by activated endothelial cells from uninjured blood vessels. The new blood vessels differentiate into arteries and venules by recruitment of pericytes and smooth muscle cells [9]. Re-epithelialization is essential for the re-establishment of tissue integrity, which is ensured by local keratinocytes at the wound edges and epithelial stem cells from skin appendages such as hair follicles or sweat glands [10]. Granulation tissue formation is the last step in the proliferation phase, characterized by accumulation of a high density of fibroblasts, granulocytes, macrophages, capillaries and collagen bundles, which replace the provisional wound matrix formed during the inflammation stage. The predominant cells in this tissue are fibroblasts, which produce types I and III collagen and ECM substances, providing a structural framework for cell adhesion and differentiation [11]. Later, myofibroblasts induce wound contraction by virtue of their multiple attachment points to collagen and help to reduce the surface area of the scar [12].

The remodeling stage is already initiated while the granulation tissue is formed. During the maturation of the wound, type III collagen, which was produced in the proliferation stage, is replaced by the stronger type I collagen which is oriented as small parallel bundles and contributes to the basket-weave collagen formation in normal dermis [13].

### HTS formation

The physiological process of normal wound healing will not result in HTS formation. However, if abnormalities occur during the wound healing process, the delicate balance of ECM degradation and deposition will be disrupted. Either insufficient degradation and remodeling of ECM due to an imbalance in expression of matrix metalloproteinases (MMPs) [14] or excessive ECM deposition caused by increased activity of fibroblasts and myofibroblasts [15] might lead to HTS formation. One common mechanism that burn patients often end up with HTS formation is the chronic inflammation or infection due to the severity of the injury, which prolongs the wound healing process and leads to excessive scarring [16]. This prolonged inflammatory phase will lead to HTS formation such as increased vessel and cell number as well as excessive collagen deposition [17].

It is well accepted that fibroblasts and myofibroblasts play essential roles in fibrotic diseases due to their abilities to generate excessive collagen in abnormal wound healing conditions [18, 19]. However, growing evidence suggests that other cells actively participate in scar pathogenesis, for example, keratinocytes and mast cells [20, 21]. When co-cultured with keratinocytes, fibroblasts exhibited significant proliferation activity [22]. The proliferation of dermal fibroblasts can also be stimulated by intercommunication of epidermal keratinocytes while decreasing the collagen production [23]. The activated keratinocytes in HTS tissue showed abnormal epidermal-mesenchymal interactions due to delayed re-epithelialization and prolonged epidermal inflammation, indicating that abnormal wound healing such as severe burn injuries may end up with HTS formation because the regulation of keratinocytes to fibroblasts is impaired [24]. However, independently co-culturing layered fibroblasts and keratinocytes on collagen-glycosaminoglycan scaffolds, aiming to assess the influence of keratinocytes and layered fibroblasts on the characteristics of tissue-engineered skin, showed that keratinocytes reduced fibrotic remodeling of the scaffolds by deep dermal fibroblasts, demonstrating an anti-fibrotic role of keratinocytes on layered fibroblasts in a 3D microenvironment [25]. In addition mast cells appear to activate fibroblasts through gap junction intercellular communication (GJIC), indicating that mast cell-fibroblast GJIC may also play a role in fibrosis [26]. Eliminating the mast cell or its GJIC with fibroblasts may prevent HTS formation or reduce the severity of fibrosis [27]. Mast cells are able to stimulate the proliferation of fibroblasts by releasing biological mediators such as histamine, chymase and tryptase via degranulation, which leads to the promotion of fibrogenesis [28, 29]. Additionally, histamine is able to enhance the effect on fibroblast migration and proliferation in vitro [30]. More histamine was found in HTS mast cells compared to normal skin mast cells after stimulation by a neuropeptide, substance P [31]. In an in vivo experiment, histamine was found significantly elevated in the plasma of patients with HTS compared to age-matched normal volunteers [32]. The elevated histamine can cause vasodilation and itchiness, resulting in the typical pruritic behavior that severely affects patients with HTS [33].

### Differences between HTS and keloids

HTS and keloids are both caused by abnormal wound healing and are characterized by pathologically excessive fibrosis in the skin [34]. Sometimes the differentiation between HTS and keloids can be difficult and lead to incorrect identification, which may result in inappropriate treatment [35].

HTS are mostly caused by trauma or burn injury to the deep dermis and do not extend beyond the boundary of the original injury. Keloids can develop after minor injuries and may even spontaneously form on the sternal region without obvious injury, which will project beyond the original wound borders [36, 37]. HTS are red, raised and mostly linear scar occurred in any regions of the body while keloids appear as pink to purple, shiny, rounded protuberances and are commonly seen in sternal skin, shoulder, upper arms and earlobe. HTS usually appear within a few months of injury, regress in one or a few years and can cause contracture when joint regions are affected, whereas, keloids might take years to develop, grow for years and do not cause contracture. Keloids are commonly seen in darker skin population and have never been reported in albino populations [38].

HTS are characterized by abundant alpha-smooth muscle actin (α-SMA) producing myofibroblasts together with more type III collagen than type I collagen. On the contrary, there is no α-SMA producing myofibroblasts and a mixture of type I and type III collagen is found in keloid tissue [34]. The collagen bundles in keloids are thick, large and closely packed random to epidermis, whereas fine, well-organized parallel to epidermis collagen bundles are found in HTS [39]. ATP in keloids remained at higher levels for a long time while ATP level decreased over time in HTS [40]. An investigation of the expression of three proteins of the p53 family in keloids and HTS showed that the level of p53 proteins was higher in keloids compared to HTS. Protein p73 was elevated only in HTS and no difference was found between keloids and HTS of the level of p63 [41]. An in vitro analysis of ECM contraction by fibroblasts isolated from different scars showed that HTS fibroblasts had a consistently higher basal level of fibrin matrix gel contraction than keloid fibroblasts [42]. Despite all these differences, HTS and keloids possess similar features including excessive ECM deposition such as high collagen content and rich proteoglycan levels within the dermis and subcutaneous tissue [43]. The treatment for HTS and keloids are similar but HTS has a better prognosis for surgical excision because keloids have a much higher recurrence rates [16].

### Complications of HTS

Complications of HTS include pain, pruritus, immobility of joint region, disfigurement and psychological issues. Pain and pruritus might not be as devastating as other complications, but they are significant complaints for many patients with HTS and they have been shown to persist for decades. The pain patients with HTS experience is often neuropathic pain, which is caused by dysfunction in the peripheral or central nervous system due to the primary injury. The neuropathic pain symptoms complained by patients with HTS are pins and needles, burning, stabbing, shooting or electric sensations [44]. The mechanism of pruritus is not well understood, but it is associated with histamine, which is released by mast cells and implicated as a primary mediator of itchiness [45]. Patients who developed HTS also suffer from reduced functional range of motion due to joint contractures, and disfigurement due to HTS tissue formed in the visible area of the body, which can lead to psychological problems or even social issues. A cross-sectional descriptive study showed that patients with HTS suffered from pain, joint stiffness, handicaps in walking or running up to on average of 17 years since the severe burn injury [46]. With all these complications, patients with HTS have complicated psychiatric disorders, including concern of body image, anxiety, depression, low self-esteem and posttraumatic stress. They have needs for psychological counseling and rehabilitation, especially for those who are economically disadvantaged or with preexisting mental illness [47]. However, a study focused on adolescent with disfiguring burn scars showed that instead of viewing themselves as less personally competent than unburned adolescents, they exhibited a similar or higher degree of self-worth as compared to their peers [48].

### Treatments of HTS

The outcome of HTS is quite different because of the varied injured sites, severity of the injuries, and treatments the patients receive which leads to a variety of therapeutic strategies between surgeons and hospitals [49]. The effect of current treatment of HTS is slow and incomplete while being expensive, time consuming and labor intensive. In 2002, Mustoe et al. reported a qualitative overview of the available clinical literature by an international advisory panel of experts and provided evidence-based recommendations on prevention and treatment of HTS, which was considered as an outline for scar management [50]. Surgical excision combined with adjuvant therapies such as steroids, pressure garments and silicone gel is still the most common current management [51]. There are similar studies published in 2014 by Gold et al. [52, 53], which tried to standardize scar management by establishing safe and effective treatment options in order to apply in routine clinical practice. They conducted a comprehensive search of the MEDLINE database over the past 10 years and suggested that the most significant advances were laser therapy [54] and 5-fluorouracil [55]. Emerging therapies for HTS were also reported such as bleomycin [56], onion extract gel [57, 58], and Botulinum toxin A [59].

## Review

### Molecular basis of HTS

#### Cytokines in HTS formation

##### Interleukin-1 alpha (IL-1α) and TNF-α inhibit HTS

Interleukin-1 (IL-1) has two subtypes, IL-1α and IL-1β. IL-1α was found to promote the release of MMPs, activate MMP-1 and stimulate the degradation of ECM [60, 61]. Thus, decreased levels of IL-1α may lead to ECM accumulation and HTS. The expression of IL-1α was found significantly lower in HTS than in normal skin from patients following breast reduction surgery [62]. Quite different from IL-1α, IL-1β is found to be over-expressed in HTS compared to normal skin [63].

TNF-α participates in the early inflammation stage and the ECM remodeling phase. TNF-α is also believed to cause fibrosis together with IL-1β [64]. However, TNF-α expression was shown to be decreased in HTS compared to normal skin, which indicated that TNF-α may be important for wound healing and HTS might be partially a consequence of a decreased amount of TNF-α [65]. Another experiment demonstrated that TNF-α could suppress transforming growth factor beta-1 (TGF-β1)-induced myofibroblasts phenotypic genes such as α-SMA at the mRNA level as well as at the Smad signaling pathway of TGF-β1 [66].

##### Inappropriate release of IL-6 leads to HTS

IL-6 is also involved in the wound healing process. It is one of the major regulators of cells stimulation, angiogenesis and ECM synthesis [67]. IL-6 could also cause fibrotic diseases such as pulmonary fibrosis and scleroderma [68, 69]. In addition, IL-6 was reported to be highly expressed in fibroblasts from HTS tissue compared to normal fibroblasts, influencing scar formation by modulating fibroblasts [70]. In order to further investigate the function of IL-6, fibroblasts from HTS were treated with IL-6. Results showed an absence of any up-regulation of MMP-1 and MMP-3, indicating that suppression of MMPs may play a role in the excessive accumulation of collagen formed in HTS [71]. In fetal fibroblasts, there was less IL-6 produced compared to adult fibroblasts and the addition of exogenous IL-6 caused scar formation instead of scarless wound healing [72]. However, IL-6 knock-out mice showed delayed wound healing [73].

##### Interleukin-10 (IL-10) plays an important role in scarless wound healing by regulating pro-inflammatory cytokines

IL-10 is produced by T helper cells and it could mediate the growth or functions of various immune cells including T cells and macrophages. It has been established that IL-10 acts as a key anti-inflammatory cytokine, which could limit or terminate the inflammatory processes [74]. Neutralizing antibodies of IL-10 were administered into incisional wounds in mice and the results demonstrated an inhibited infiltration of neutrophils and macrophages and an over-expression of monocyte chemotactic protein-1 (MCP-1), IL-1β, TNF-α [75] and IL6 [76]. This is supported by another study that IL-10 significantly inhibited lipopolysaccharide (LPS)-induced IL-6 production at a transcriptional level [77]. A study tried to evaluate whether IL-10 could change the innervated conditions of full thickness excisional wounds created on the dorsal surface of CD1 mice. The results showed only temporary changes during the wound healing process but no significant changes at 84 days after treatment. However, wounds treated with IL-10 recovered similarly to normal skin compared to the wounds treated with PBS [78]. Another experiment reported that scar appeared in IL-10 knockout fetal mice compared to scarless wound healing in the control group [79]. A more recent study showed that IL-10 could provide an optimal environment for fetal and postnatal scarless wound healing [80]. A similar study also over-expressed IL-10 but in adult murine wounds. The results showed that increased IL-10 reduced inflammation, collagen deposition and created improved wound healing conditions [81].

#### Growth factors in HTS formation

##### Transforming growth factor-β (TGF-β) plays a pivotal role in HTS formation

TGF-β is one of the most important growth factors that regulate tissue regeneration, cell differentiation, embryonic development and regulation of the immune system [82–84]. Recent studies showed that TGF-β not only involves in normal wound healing process but also contributes to fibroproliferative disorders such as pulmonary fibrosis [85] and HTS [86]. TGF-β has three isoforms, TGF-β1, transforming growth factor-beta 2 (TGF-β2) and transforming growth factor-beta 3 (TGF-β3) [87]. Shah et al. used the neutralizing antibody to TGF-β1 and TGF-β2 in cutaneous wounds of adult rodents and found reduced cutaneous scarring formation [88]. A subsequent study from Shah reported that exogenous addition of TGF-β3 to cutaneous rat wounds reduced scarring, indicating that TGF-β1 and TGF-β2 were related to cutaneous scarring while TGF-β3 should be considered as a therapeutic agent against scarring [89]. A more recent study treated the rabbit ear wounds with anti-TGF-β1, 2, 3 monoclonal antibodies at different time points of wound healing and early injection of antibodies showed delayed wound healing while the injections of middle or later time points remarkably reduced HTS formation, which implicated the indispensable roles of TGF-β1, 2, 3 in early stage of wound healing [90]. The transcriptional factor forkhead box protein O1 (FOXO1) has recently been found to be important as a regulator in wound healing. It exerts its effect through regulation of TGF-β1 expression from oxidative stress. The absence of FOXO1 reduced TGF-β1 expression and led to impaired re-epithelialization of wounds [91].

Many studies indicate that aberrant TGF-β expression plays a pivotal role in HTS formation. For example, a previous study showed that the serum level of TGF-β1 was up-regulated locally and systemically in burn patients and a significant clinical improvement in scar quality and volume was obtained after interferon-alpha2b (IFN-α2b) therapy, which was associated with normalization of serum TGF-β1 [92]. Treatment of IFN-α2b and interferon-gamma (IFN-γ) to site-matched HTS and normal fibroblasts showed antagonized TGF-β1 protein production, down-regulation of TGF-β1 mRNA levels [93]. Tredget et al. made superficial partial-thickness ear wound and full-thickness back wounds on a transgenic mouse over-expressing TGF-β1 in order to investigate the endogenous derived TGF-β1 on wound re-epithelialization. The findings suggested that over-expression of TGF-β1 speeded the rate of wound closure in partial-thickness wounds; whereas, over-expression of TGF-β1 slowed the rate of wound re-epithelialization in full-thickness wounds [94]. Another study created superficial and deep horizontal dermal scratch experimental wounds on the anterior thigh of adult male patients in order to characterize the related expression of TGF-β1 and TGF-β3. HTS formed after injuries to the deep dermis while superficial wounds healed with minimal or no scarring. Higher TGF-β1 and lower TGF-β3 expression was found in deep wounds compared to superficial wounds, suggesting the pivotal role of TGF-β1 in HTS formation [95].

##### Connective tissue growth factor (CTGF) acts as a downstream mediator of TGF-β1 signaling pathway and involves in HTS formation

CTGF, also know as CCN2, is a pleiotropic cytokine that is induced by TGF-β1 in dermal fibroblasts and is considered to be a downstream mediator of TGF-β1 [96]. The main role of CTGF is to interact with signaling proteins such as TGF-β1 for the regulation of cell proliferation, differentiation, adhesion, ECM production and granulation tissue formation [97, 98]. This collaboration between CTGF and TGF-β1 has contributed to the pro-fibrotic properties of TGF-β1 confirming the role of CTGF for TGF-β1 induction as a co-factor of gene expression.

The expression of CTGF was found increased in cultured fibroblasts from HTS, keloids and chronic fibrotic disorders [99]. In addition, cultured fibroblasts from HTS showed an increased expression of CTGF after stimulation by TGF-β [100]. In order to evaluate the role of CTGF in HTS formation, a rabbit animal model was established by Sisco at el. Antisense therapy was used to inhibit the expression of CTGF. Real-time reverse transcription polymerase chained reaction demonstrated an increased expression of CTGF in scar tissue and decreased CTGF expression after the intradermal injection of antisense oligonucleotides. The study showed that inhibition of CTGF in different times in wound healing has a substantial effect on reducing HTS [101]. Another experiment used CTGF small interfering RNA (siRNA) to successfully block the increase in CTGF mRNA levels and the result demonstrated that CTGF could regulate the gene expression of ECM, tissue inhibitor metalloproteinases and partial function of TGF-β1 [102]. In order to elucidate the pathophysiological function of CTGF, CTGF knock-out mice were used in the experiment and those mice died immediately after birth due to malformation of the rib cages. As well, the embryonic fibroblasts from this animal model showed an inability of adhesion and α-SMA formation. All these results suggest that CTGF functions in ECM adhesion and production [103, 104].

Taken together, CTGF acts as a downstream mediator of the TGF-β1 signaling pathway, directly involved in ECM synthesis and assists with TGF-β1 in the pathogenesis of HTS.

##### Platelet-derived growth factor (PDGF) is essential to wound healing and the over-expression of PDGF is important in the formation of HTS

PDGF has five isoforms, including PDGF-AA, PDGF-AB, PDGF-BB, PDGF-CC and PDGF-DD which function via the activation of three transmembrane receptor tyrosine kinases (RTKs) [105]. PDGF is produced by degranulated platelets in the early phase of the wound healing process and it is also secreted by macrophages during the proliferative phase of wound healing [106]. In wound healing-impaired mice, the expression of PDGF and their receptors decreased [107]. Moreover, PDGF showed reduced expression in chronic human non-healing ulcers compared to the fresh surgically created acute wounds [108]. All these studies support the important role of PDGF in wound healing. However, PDGF also has an important role in several fibrotic diseases including scleroderma, lung and liver fibrosis by promoting the growth and survival of myofibroblasts [109]. PDGF was found to mediate the deposition of collagen in fibroblasts and it was highly over-expressed in both the epidermis and the dermis of HTS. Over-production of collagen was not only related to high levels of TGF-β1, but also with increased expression of PDGF [110]. Another experiment showed that PDGF stimulated myofibroblast formation and increased TGF-β receptor I (TGF-βRI) and TGF-β receptor II (TGF-βRII) expression [111].

Although there are a lot of studies showing that PDGF plays a role in the pathogenesis of HTS, the exact molecular mechanism is still unknown.

##### Inhibitory effect of basic fibroblast growth factor (bFGF) on HTS via the regulation of collagen production, myofibroblast differentiation and TGF-β receptor expression

Fibroblast growth factors (FGFs) are a large family of growth factors that consist of 22 members with similar structural polypeptide. They have four receptors, which are transmembrane protein tyrosine kinases [112, 113]. Among the growth factors that play roles in wound healing, bFGF is particularly important [114]. bFGF is produced by keratinocytes and is found in the early stages of wound healing. It stimulates growth and differentiation of several types of cells, such as fibroblasts [115]. In a rat model, bFGF was detected in granulation tissue including regenerated epidermis and newborn capillaries [116]. As well, bFGF was found to promote wound healing by stimulating angiogenesis and granulation tissue proliferation [117]. However, bFGF might inhibit the granulation tissue formation by promoting apoptosis [118] and affect tumor growth [119].

Evidence for the importance of bFGF in the pathogenesis of HTS was provided by Tiede et al. that bFGF reduced α-SMA expression by inhibiting myofibroblast differentiation and it also decreased TGF-βRI and TGF-βRII expression [111]. In a rabbit HTS ear model, bFGF was applied everyday for three months and the wounds showed decreased collagen expression and increased MMP-1 expression such that bFGF appeared to have a negative effect on scar formation [120]. In humans bFGF was administered to acute incisional wounds after suturing and the patients remained free from HTS [121]. Hepatocyte growth factor (HGF) and MMP-1 have been demonstrated to have an anti-scarring effect [122]. In a more recent study, the expressions of HGF and MMP-1 were highly regulated in bFGF treated HTS and normal fibroblasts. The highly regulated MMP-1 expression might contribute to the increase of type I and type III collagen degradation, which leads to reduced scar formation. In vitro, bFGF treatment significantly decreased scar weight and the amount of collagen in nude mice that underwent human scar tissue transplantation [123]. Therefore, bFGF can inhibit HTS formation and the mechanism might be related to the regulation of collagen production, myofibroblast differentiation and inhibition of TGF-β receptor expression.

#### Macrophages involve in HTS formation via Stromal cell-derived factor 1 (SDF-1)/CXCR4 chemokine pathway

Significant more mast cells, fibrocytes and macrophages were found in nude mice that received human split thickness skin graft (STSG) compared to nude mice that received human full thickness skin graft (FTSG) in vivo, where HTS formation was found on both mice 2 months after the grafting with more scar observed in mice that received STSG, suggesting that inflammatory cells and bone marrow-derived fibrocytes might play critical roles in HTS formation in this human HTS-like nude mouse model [124]. A sequent study showed increased grafted skin thickness, increased number of myofibroblasts, decreased decorin and increased biglycan expression, positive staining of human leukocyte antigen in STSG grafted skin that formed persistent scars, which showed morphologic, histologic and immunohistochemical consistency with human HTS [125]. This animal model provides a means to study HTS and test new novel treatment options. Although there is not an ideal animal model that can be directly translated into human subjects to clearly explain the molecular basis of HTS formation, the human HTS-like nude mouse model is closer to the perfect animal model because the survived human skin grafts possess the genetic and histological properties of human HTS.

SDF-1 is found to be a potent chemokine that attracts lymphocytes and monocytes by binding exclusively to its receptor, CXCR4 [126–128]. Studies focused on the functions of SDF-1/CXCR4 signaling have suggested that it involves not only in the tumor metastasis and vascularization but also in the pathogenesis of fibroproliferative diseases [129, 130]. Recent studies found up-regulated SDF-1 expression in the HTS tissue and serum of the burn patients as well as increased number of CD14+ CXCR4+ cells in the peripheral blood mononuclear cells, which suggested that SDF-1/CXCR4 signaling could recruit these CXCR4+ cells such as monocytes to the prolonged inflamed injured site and contribute to HTS formation [131]. In order to further verify the role of SDF-1/CXCR4 signaling in HTS formation, the CXCR4 antagonist CTCE-9908 was used to inhibit the SDF-1/CXCR4 effect on the human HTS-like nude mouse model. The study showed that CTCE-9908 significantly attenuated scar formation and contraction, reduced the number of macrophages in the tissue, which was differentiated and replenished by CXCR4 expressing monocytes in the circulation [132]. These findings support the role of SDF-1/CXCR4 in HTS formation and suggest an important role of macrophages in HTS formation.

Macrophages were first discovered by a Russian scientists, Élie Metchnikoff, in 1884 [133]. They are differentiated from newly recruited monocytes from the circulation. They are considered to play a vital role in the whole wound healing process because recent studies showed that impaired wound healing was associated with decreased number of macrophage infiltration at the injured site [134, 135]. However, pathological functioning of macrophages in the abnormal wound healing process can lead to disordered wound healing, including the formation of HTS [136]. Macrophages have two phenotypes, classically activated macrophages or the so called M1 macrophages and alternatively activated macrophages or the so called M2 macrophages [137]. Mahdavian et al. reported that M1 and M2 macrophages have distinct opposite functions in the wound healing process [136]. M1 macrophages can induce MMP-1 secretion and promote ECM degradation while M2 macrophages can secret large amount of TGF-β1, which can stimulate myofibroblast transformation and lead to ECM deposition. It is also hypothesized that prolong inflammatory phase will attract more macrophages and those macrophages will initially be more pro-inflammatory M1 phenotype and then switch to a more pro-fibrotic M2 phenotype due to more intense stimuli from the microenvironment [138]. The most distinct difference between M1 and M2 macrophages is that in M1 macrophages the arginine metabolism is shifted to nitric oxide and citrulline while in M2 macrophages it is shifted to ornithine and polyamines [139]. Growing evidence suggests that M2 macrophages are not constituted by a uniform population but can be further subdivided into M2a, M2b and M2c subsets [140]. M2a macrophages are induced by IL-4 and IL-13, which are involved in the anti-parasitic immune response and are considered to be pro-fibrotic. M2b macrophages are induced by IL-1β, LPS and immune complexes while M2c macrophages are induced by IL-10, TGF-β and glucocorticoids [141]. The fourth type, M2d macrophages, are characterized by switching from a M1 phenotype into an angiogenic M2-like phenotype, which termed M2d by Leibovich et al. [142].

**Fig. 2:**
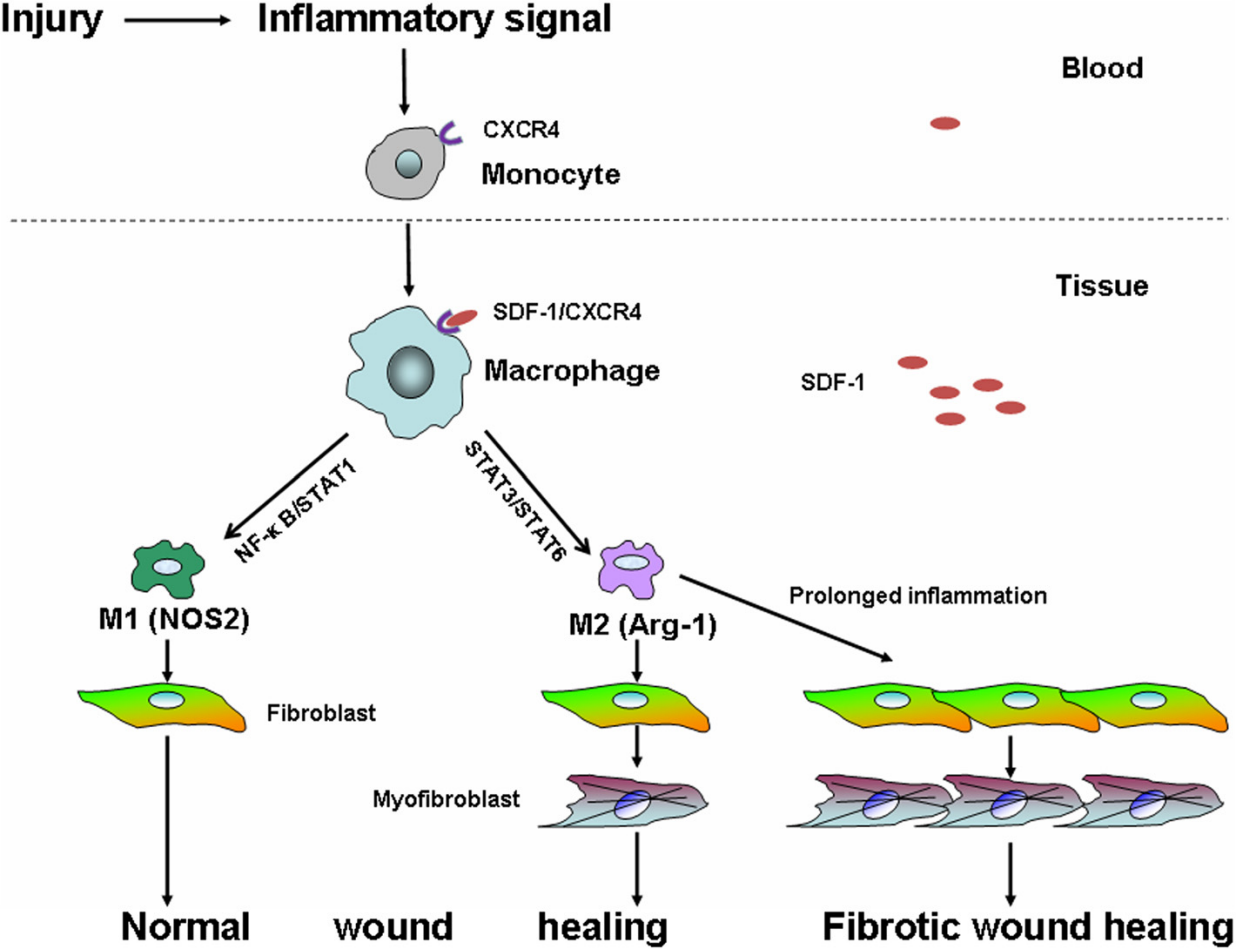
The roles of monocytes and polarized macrophages in HTS formation. We hypothesize that monocytes in the blood are recruited to the injured site via the SDF-1/CXCR4 signaling pathway and differentiate into polarized macrophages. The polarized M1 and M2 macrophages then exert their functions via various signaling pathways and involve in wound healing and HTS formation

Although studies suggest a close relationship between SDF-1/CXCR4 signaling and macrophage infiltration in the formation of HTS, more studies on the interaction between the two is still needed. Meanwhile, the roles of macrophage phenotypes in different phases of abnormal wound healing, like HTS-like nude mouse model, are to be investigated. Here we hypothesize that the monocytes, CXCR4 expressing cells in the circulation, will be attracted to the injured site via the SDF-1/CXCR4 signaling pathway due to concentration difference between the circulation and local tissue as well as the chemotactic effect of SDF-1. The monocytes then differentiate into M1 macrophages (NF-κB and STAT1 signaling pathways) and M2 macrophages (STAT3 and STAT6 signaling pathways) [143]. M1 macrophages secret pro-inflammatory cytokines such as IFN-γ, IL-1β, TNF-α, IL-6, IL-8 and generate reactive oxygen and nitric oxide through the activation of nitric oxide synthase 2 (NOS2). On the other hand, M2 macrophages inhibit the NOS2 activity via the activation of arginase-1. The distinct opposite and complementary functions of M1 and M2 macrophages will eventually lead to normal wound healing. However, in prolonged inflammatory environment such as wounds from a patient who suffered from severe thermal injury, large amounts of TGF-β1 can be produced together with increased myofibroblast proliferation, which will result in ECM deposition and finally HTS formation (Fig. 2).

## Conclusions

In this review, four phases of normal wound healing are discussed before outlining the pathogenesis of HTS, illustrating the delicate balance of ECM deposition and degradation which influences the outcome of the wound healing process. Differentiating HTS from keloids is also important because the clinical and molecular mechanisms are different leading to distinct therapeutic outcomes. HTS formation is a dynamic, complex process that involves interactions between multiple factors such as inflammatory cells, cytokines, growth factors, and chemokines. Keratinocytes and mast cells are considered to be involved in HTS formation. The role of cytokines such as IL-1, TNF-α, IL-6 and IL-10 as well as growth factors such as TGF-β, CTGF, PDGF and bFGF in HTS formation were discussed. Despite the complexity of HTS, more attentions are drawn to the molecular and cellular mechanism of HTS for technological and scientific advances such as the establishment of new animal models and in vitro techniques. Growing studies are focusing on the roles of polarized macrophages in HTS formation and it is suggested that polarized macrophages actively participate in HTS formation via the SDF-1/CXCR4 signaling pathway. A preliminary experiment conducted by our laboratory confirmed potential roles of M2 macrophages in HTS formation. A subsequent study of specific depletion of M2 macrophages by Cre-LoxP technology on our human HTS-like nude mouse model together with the study of the roles of molecular precursors mentioned above might provide novel findings and potential new treatment and prevention of HTS.

## Abbreviations

α-SMA: Alpha-smooth muscle actin
bFGF: Basic fibroblast growth factor
CTGF: Connective tissue growth factor
ECM: Extracellular matrix
FGFs: Fibroblast growth factors
FOXO1: Forkhead box protein O1
FTSG: Full thickness skin graft
GJIC: Gap junction intercellular communication
HGF: Hepatocyte growth factor
HTS: Hypertrophic scars
IFN-α2b: Interferon-alpha2b
IFN-γ: Interferon-gamma
IL-1: Interleukin-1
IL-1α: Interleukin-1 alpha
IL-1β: Interleukin-1 beta
IL-6: Interleukin-6
IL-10: Interleukin-10
LPS: Lipopolysaccharide
MCP-1: Monocyte chemotactic protein-1
MMPs: Matrix metalloproteinases
NOS2: Nitric oxide synthase 2
PDGF: Platelet-derived growth factor
PMNs: Polymorphonuclear neutrophils
RTKs: Receptor tyrosine kinases
SDF-1: Stromal cell-derived factor 1
siRNA: Small interfering RNA
STSG: Split thickness skin graft
TGF-β: Transforming growth factor-beta
TGF-β1: Transforming growth factor-beta 1
TGF-β2: Transforming growth factor-beta 2
TGF-β3: Transforming growth factor-beta 3
TGF-βRI: TGF-β receptor I
TGF-βRII: TGF-β receptor II
TNF-α: Tumor necrosis factor-alpha
VEGF: Vascular endothelial growth factor
